# Alternative autophagy dampens UVB-induced NLRP3 inflammasome activation in human keratinocytes

**DOI:** 10.1016/j.jbc.2024.107173

**Published:** 2024-03-17

**Authors:** Tatsuya Hasegawa, Saori Noguchi, Masaya Nakashima, Masashi Miyai, Makiko Goto, Yuko Matsumoto, Satoru Torii, Shinya Honda, Shigeomi Shimizu

**Affiliations:** 1 Shiseido Global Innovation Center, Yokohama, Japan; 2 Department of Pathological Cell Biology, Medical Research Institute, Tokyo Medical and Dental University, Tokyo, Japan

**Keywords:** alternative autophagy, inflammasome, keratinocyte, skin, sunburn

## Abstract

Sunlight exposure results in an inflammatory reaction of the skin commonly known as sunburn, which increases skin cancer risk. In particular, the ultraviolet B (UVB) component of sunlight induces inflammasome activation in keratinocytes to instigate the cutaneous inflammatory responses. Here, we explore the intracellular machinery that maintains skin homeostasis by suppressing UVB-induced inflammasome activation in human keratinocytes. We found that pharmacological inhibition of autophagy promoted UVB-induced NLRP3 inflammasome activation. Unexpectedly, however, gene silencing of Atg5 or Atg7, which are critical for conventional autophagy, had no effect, whereas gene silencing of Beclin1, which is essential not only for conventional autophagy but also for Atg5/Atg7-independent alternative autophagy, promoted UVB-induced inflammasome activation, indicating an involvement of alternative autophagy. We found that damaged mitochondria were highly accumulated in UVB-irradiated keratinocytes when alternative autophagy was inhibited, and they appear to be recognized by NLRP3. Overall, our findings indicate that alternative autophagy, rather than conventional autophagy, suppresses UVB-induced NLRP3 inflammasome activation through the clearance of damaged mitochondria in human keratinocytes and illustrate a previously unknown involvement of alternative autophagy in inflammation. Alternative autophagy may be a new therapeutic target for sunburn and associated cutaneous disorders.

The skin is the largest organ of the body, and the epidermis, which is the outermost layer of the skin, regulates the influx and efflux of water, electrolytes, and lipids, as well as blocking the entry into the body of xenobiotics and microbial pathogens (1, 2). However, UV radiation can penetrate the epidermis, and exposure to UV radiation, especially the UVB (wavelength range 280–320 nm) component of sunlight, can result in DNA damage and a cutaneous inflammatory reaction commonly known as sunburn, which increases skin cancer risk (2). Approximately, two billion incidents of sunburns have been estimated to occur each year (3). Furthermore, mutations are accumulated even in normal sun-exposed skin, without necessarily affecting the physiological functions of the epidermis (4, 5). Constant exposure to solar radiation places unique pressure on the repair and protection mechanisms in our skin compared to other organs. However, the nature of these mechanisms, in particular the intracellular machinery through which epidermal homeostasis is restored after UV radiation, remain unclear.

Inflammasomes are cytosolic molecular platforms that sense pathogens and cellular damage and facilitate the secretion of interleukin (IL)-1β and IL-18 to instigate inflammatory responses, thereby serving as regulators of the innate immune system (6, 7, 8). Although inflammasomes are critical for defense against bacteria and virus infections, stringent regulation of their activation is indispensable to prevent inappropriate inflammatory responses. This is particularly important in the case of NLRP3 inflammasomes, which are activated in response to not only foreign pathogens but also host self-derived metabolites. NLRP3 inflammasomes have been implicated in the chronic sterile inflammation commonly found in metabolic disorders, neuronal diseases, and age-related degenerative disorders (9, 10, 11, 12). Further, NLRP3 inflammasomes can also be activated by a broad range of environmental stimuli, including nanoparticles (13) and UV radiation (14, 15). Therefore, it is important to identify the regulatory mechanisms that limit UV-induced NLRP3 inflammasome activation in order to block excessive inflammatory responses in the skin.

Macroautophagy (hereafter referred to as autophagy) is an evolutionally conserved intracellular degradation and recycling system (16, 17). During mammalian autophagy, cytoplasmic constituents, such as proteins, nucleic acids, organelle, and invading bacteria, are enveloped by autophagosomes, and then the autophagosomes fuse with lysosomes to degrade the sequestered cargos (17). This lysosomal pathway of autophagy plays a crucial role in cellular homeostasis through the removal of detrimental cargos and the removal or recycling of degraded or dysfunctional cellular components (17). Autophagy thus contributes to protection against numerous diseases, including metabolic disorders, neuronal diseases, infections, cancer, and aging (18). Various inflammatory diseases are also demonstrated to be caused by autophagy dysfunction. For example, deficiencies in autophagy-related genes, such as *Atg5* or *Atg7*, cause aberrant inflammasome activation, leading to progression of metabolic disorders and neuronal diseases, such as atherosclerosis (19) and Alzheimer’s disease (20).

Atg5 and Atg7 are thought to be essential for autophagy (21), but recent findings indicate that Atg5- or Atg7-deficient cells still form autophagosomes and autolysosomes to eliminate proteins and organelles (22, 23, 24). Thus, mammalian autophagy can occur *via* at least two different pathways, the Atg5/Atg7-dependent conventional pathway and an Atg5/Atg7-independent alternative pathway (also called Golgi-mediated degradation), in a stimulus-dependent and context-dependent manner (22, 25). The mechanisms and physiological roles of conventional autophagy have been well studied using Atg5-or Atg7-deficient cells, whereas those of alternative autophagy have not been fully elucidated yet (26).

In this study, we show that UVB-induced NLRP3 inflammasome activation is suppressed in part through the clearance of damaged mitochondria in human keratinocytes via Atg5/Atg7-independent alternative autophagy, rather than conventional autophagy. This is the first report to demonstrate the involvement of alternative autophagy in inflammation.

## Results

### Autophagy suppresses UVB-induced NLRP3 inflammasome activation in human keratinocytes

**Figure 1:**
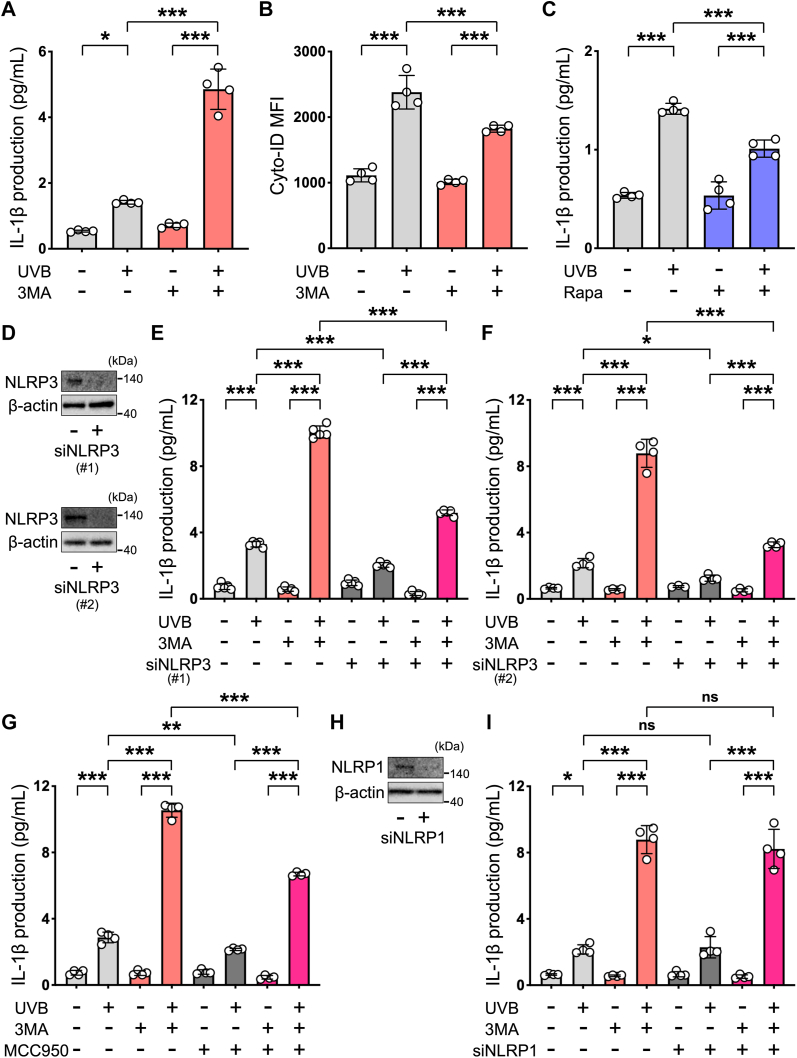
**Autophagy suppresses UVB-induced NLRP3 inflammasome activation in human keratinocytes.***A*, quantification of IL-1β production by ELISA in the supernatants of keratinocytes, which were incubated for 24 h in the absence or presence of autophagy inhibitor 3MA, then irradiated or not irradiated with UVB, and further cultured for 48 h (*n* = 4 per group). *B*, flow cytometric analysis to detect autophagy using Cyto-ID. Cells were incubated for 24 h in the absence or presence of autophagy inhibitor 3MA, then irradiated or not irradiated with UVB, and further cultured for 6 h (*n* = 4 per group). *C*, quantification of IL-1β production by ELISA in the supernatants of keratinocytes incubated for 24 h in the absence or presence of the autophagy inducer rapamycin (Rapa), then irradiated or not irradiated with UVB, and further cultured for 48 h (*n* = 4 per group). *D*, representative example showing the expression level of NLRP3 evaluated by Western blotting in the cell lysates of keratinocytes, which were transfected with either control siRNA or NLRP3 siRNA. *E*, quantification of IL-1β production by ELISA in the supernatants of keratinocytes, which were transfected with either control siRNA or NLRP3 siRNA and then irradiated or not irradiated with UVB and further cultured for 48 h in the absence or presence of the 3MA (*n* = 5 per group). *F*, quantification of IL-1β production by ELISA in the supernatants of keratinocytes, which were transfected with either control siRNA or NLRP3 siRNA (#2) and then irradiated or not irradiated with UVB and further cultured for 48 h in the absence or presence of 3MA (*n* = 4 per group). *G*, quantification of IL-1β production by ELISA in the supernatants of keratinocytes incubated for 24 h in the absence or presence of 3MA or NLRP3 inhibitor MCC950, then irradiated or not irradiated with UVB, and further cultured for 48 h (*n* = 4 per group). *H*, representative example showing the expression level of NLRP1 evaluated by Western blotting in the cell lysates of keratinocytes, which were transfected with either control siRNA or NLRP1 siRNA. *I*, quantification of IL-1β production by ELISA in the supernatants of keratinocytes, which were transfected with control siRNA or NLRP1 siRNA, incubated for 24 h in the absence or presence of 3MA, then irradiated or not irradiated with UVB, and further cultured for 48 h (*n* = 4 per group). All data are expressed as the mean ± SD. ∗ *p* < 0.05, ∗∗ *p* < 0.01, and ∗∗∗ *p* < 0.001, ns: not significant. IL, interleukin; UVB, ultraviolet B.

**Supporting Figure S1:**
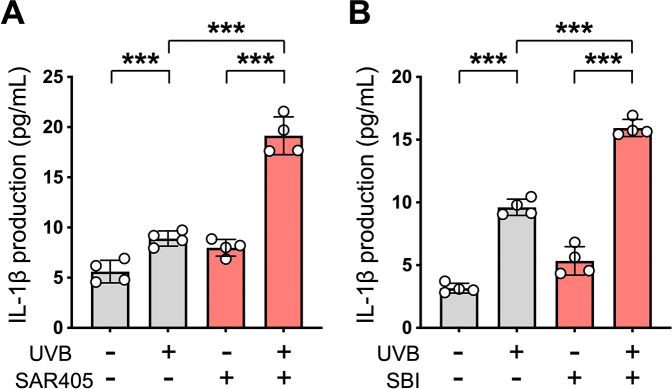


**Supporting Figure S2:**
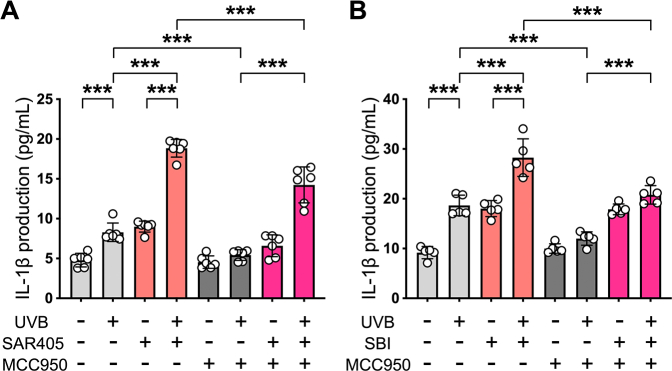


In order to explore whether autophagy regulates UVB-induced inflammasome activation in normal human epidermal keratinocytes, we evaluated changes in UVB-induced IL-1β production as a readout of inflammasome activation, following pharmacological modulation of autophagy. As shown in Figure 1*A*, UVB radiation increased IL-1β production from keratinocytes, and this production was markedly promoted in the presence of 3-methyladenine (3MA), an inhibitor of autophagy. Under this condition, we confirmed that autophagy, as monitored by Cyto-ID, was also significantly inhibited (Fig. 1*B*). In addition, different types of autophagy inhibitors, including SAR405, an inhibitor of Vps34, and SBI-0206965, an inhibitor of the autophagy initiator kinase Ulk1, also significantly promoted UVB-induced IL-1β production from keratinocytes (Fig. S1). In contrast, rapamycin, which is an autophagy inducer, significantly suppressed UVB-induced IL-1β production from keratinocytes (Fig. 1*C*), indicating the involvement of autophagy in UVB-induced inflammasome activation. The inflammasome component NLRP3 is required for UVB-induced IL-1β production from human keratinocytes, and its expression is elevated in UV-irradiated human epidermis *in vivo* (15). Next, to determine whether NLRP3 is associated with this amplification of IL-1β production under inhibition of autophagy, we prepared keratinocytes in which NLRP3 was markedly downregulated by means of siRNA treatment (Fig. 1*D*). As shown in Figure 1, *E* and *F*, knockdown of NLRP3 significantly abolished 3MA-mediated IL-1β production from UVB-irradiated keratinocytes. Furthermore, we observed that 3MA-mediated IL-1β production from UVB-irradiated keratinocytes was also inhibited by the treatment of MCC950, an inhibitor of NLRP3 (27) (Fig. 1*G*). We also confirmed that MCC950 also significantly suppressed UVB-induced IL-1β production from keratinocytes in the presence of SAR405 or SBI-0206965 (Fig. S2). On the other hand, we did not observe the influence of knockdown of NLRP1 in 3MA-mediated IL-1β production from UVB-irradiated keratinocytes under our experimental condition (Fig. 1, *H* and *I*). These findings indicate that autophagy suppresses UVB-induced NLRP3 inflammasome activation in human keratinocytes.

### Atg5/Atg7-independent alternative autophagy participates in the suppression of UVB-induced inflammasome activation in human keratinocytes

**Figure 2:**
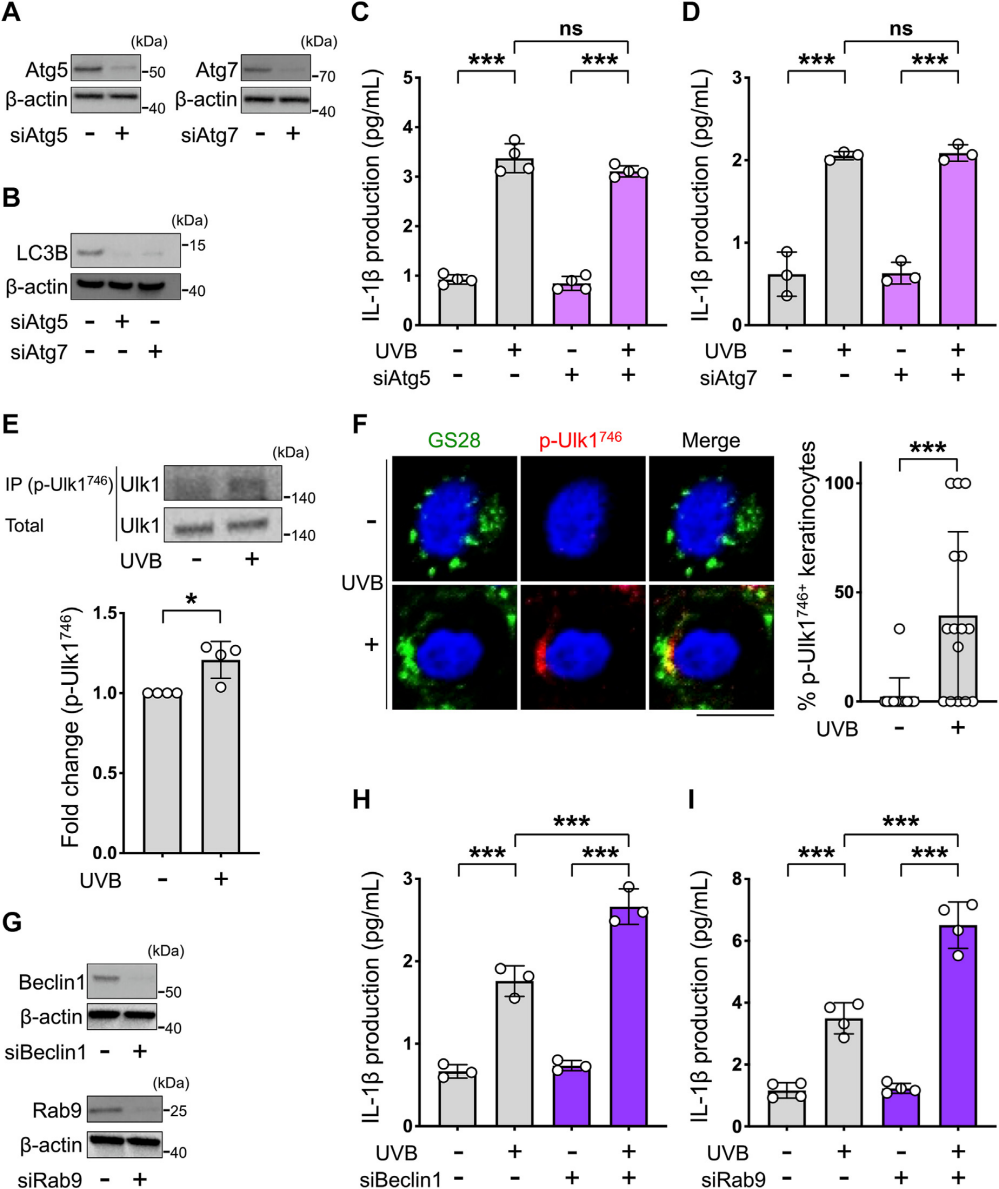
**Atg5/Atg7-independent alternative autophagy participates in the suppression of UVB-induced inflammasome activation in human keratinocytes.***A*, representative example showing the expression level of Atg5 (*left*) or Atg7 (*right*) evaluated by Western blotting in the cell lysates of keratinocytes, which were transfected with either control siRNA or Atg5 siRNA or Atg7 siRNA. *B*, representative example showing the expression level of LC3B determined by Western blotting in cell lysates of either Atg5- or Atg7-knockdown keratinocytes. *C*, quantification by ELISA of IL-1β in the supernatants of keratinocytes that were transfected with control siRNA or Atg5 siRNA for 48 h, then irradiated or not irradiated with UVB, and further cultured for 48 h (*n* = 4 per group). *D*, quantification by ELISA of IL-1β in the supernatants of keratinocytes that were transfected with control siRNA or Atg7 siRNA for 48 h, then irradiated or not irradiated with UVB, and further cultured for 48 h (*n* = 3 per group). *E*, representative example showing the expression level of p-Ulk1^746^ evaluated by immunoprecipitation-Western blotting in the cell lysates of keratinocytes, which were irradiated or not irradiated with UVB and further cultured for 6 h (*top*). Relative intensity of p-Ulk1^746^ is shown (*n* = 4 per group) (*bottom*). *F*, representative immunocytochemical staining of GS28 (*green*) and p-Ulk1^746^ (*red*) in keratinocytes, which were irradiated or not irradiated with UVB and further cultured for 6 h (*left*). The percentage of p-Ulk1^746^–positive keratinocytes. Cells were counted and averaged across 15 randomly selected images in each experiment. *n* = 15 in each group (*right*). Repeated three independent experiments were performed. *G*, representative example showing the expression level of Beclin1 or Rab9 determined by Western blotting in the cell lysates of keratinocytes transfected with either control siRNA, Beclin1 siRNA, or Rab9 siRNA. *H*, quantification by ELISA of IL-1β in the supernatants of keratinocytes that were transfected with control siRNA or Beclin1 siRNA for 48 h, then irradiated or not irradiated with UVB, and further cultured for 48 h (*n* = 3 per group). *I*, quantification by ELISA of IL-1β in the supernatants of keratinocytes that were transfected with control siRNA or Rab9 siRNA for 48 h, then irradiated or not irradiated with UVB, and further cultured for 48 h (*n* = 4 per group). Nuclei were stained with Hoechst (*blue*). All data are expressed as the mean ± SD. ∗ *p* < 0.05 and ∗∗∗ *p* < 0.001, ns: not significant. Scale bar represents 10 μm. IL, interleukin; UVB, ultraviolet B.

**Supporting Figure S3:**
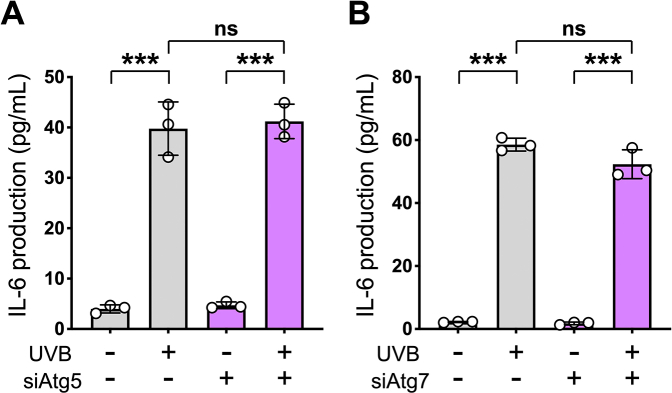


There are two types of autophagy, and Atg5 and Atg7 are essential for conventional autophagy (21). Therefore, to establish whether genetic inhibition of conventional autophagy promotes UVB-induced inflammasome activation in keratinocytes, we measured the level of UVB-induced IL-1β production by keratinocytes in which the Atg5 and Atg7 genes had been silenced with siRNA. As shown in Figure 2*A*, Atg5 and Atg7 were both markedly downregulated in the siRNA-treated keratinocytes. Consistent with this observation, the expression level of microtubule-associated protein light chain 3B, which is a standard marker of conventional autophagy, was also inhibited under silencing of either Atg5 or Atg7 (Fig. 2*B*). Unexpectedly, however, the level of UVB-induced IL-1β production was not significantly changed in either Atg5-knockdown keratinocytes (Fig. 2*C*) or Atg7-knockdown keratinocytes (Fig. 2*D*), even though conventional autophagy was inhibited in these cells. In accordance with this finding, the level of proinflammatory cytokine IL-6 was also unchanged after UVB radiation in either Atg5-knockdown keratinocytes or Atg7-knockdown keratinocytes (Fig. S3).

**Supporting Figure S4:**
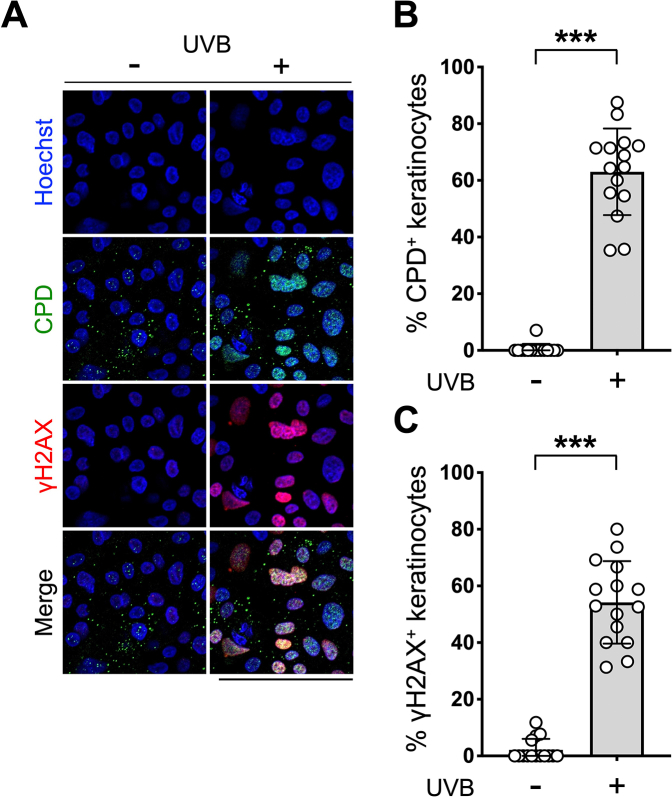


**Supporting Figure S5:**
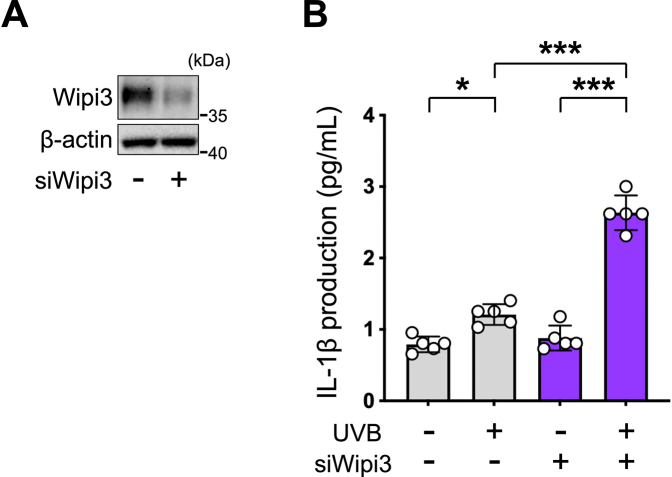


There is increasing evidence that mammalian autophagy occurs not only via the conventional Atg5/Atg7-dependent pathway but also an alternative Atg5/Atg7-independent pathway, and DNA damage induces both types of autophagy (28). In UVB-exposed keratinocytes, DNA damage was confirmed by the strong immunocytochemical staining of cyclobutane pyrimidine dimer and γH2AX (Fig. S4), which are DNA damage markers (29, 30). We thus investigated whether alternative autophagy takes place in UVB-exposed keratinocytes. For this aim, we first evaluated the cellular level of Ulk1 phosphorylation at Ser^746^, which was recently identified as a marker of alternative autophagy (31), by means of immunoprecipitation of p-Ulk1^746^ with a specific antibody (31) together with Western blotting using an anti-Ulk1 antibody. As shown in Figure 2*E*, the p-Ulk1^746^ signal was significantly increased in UVB-exposed keratinocytes. We also performed immunocytochemical staining of p-Ulk1^746^ in keratinocytes to confirm its change of expression and subcellular localization upon UVB radiation. Consistent with the immunoprecipitation-Western blotting results, we found that p-Ulk1^746^ signals were emerged after UVB radiation (Fig. 2*F*). Importantly, the signals were mainly localized at the Golgi, as assessed by GS28 immunofluorescence, where alternative autophagosomal membranes are originated (Fig. 2*F*), indicating the activation of alternative autophagy in UVB-exposed keratinocytes. Furthermore, we examined the effect of siRNA-mediated silencing of Beclin1, a molecule required for conventional autophagy and alternative autophagy (22). As shown in Figure 2, *G* and *H*, in contrast to the effect of silencing Atg5 or Atg7, the release of IL-1β from UVB-irradiated keratinocytes was significantly enhanced by silencing Beclin1. Rab9 is required for autophagosome formation from the *trans*-Golgi and late endosomes during the process of alternative autophagy, but not conventional autophagy (22). The level of UVB-induced IL-1β production was also significantly increased in Rab9-knockdown keratinocytes (Fig. 2, *G* and *I*). In addition, we confirmed that knockdown of Wipi3, which is essential for alternative autophagy but not for conventional autophagy (24, 32), significantly also promoted UVB-induced IL-1β production from keratinocytes (Fig. S5). These findings suggest that UVB induces Atg5/Atg7-independent alternative autophagy, which is partly involved in the suppression of inflammasome activation in human keratinocytes.

### Damaged mitochondria are accumulated and recognized by NLRP3 in UVB-irradiated human keratinocytes under inhibition of alternative autophagy

**Figure 3:**
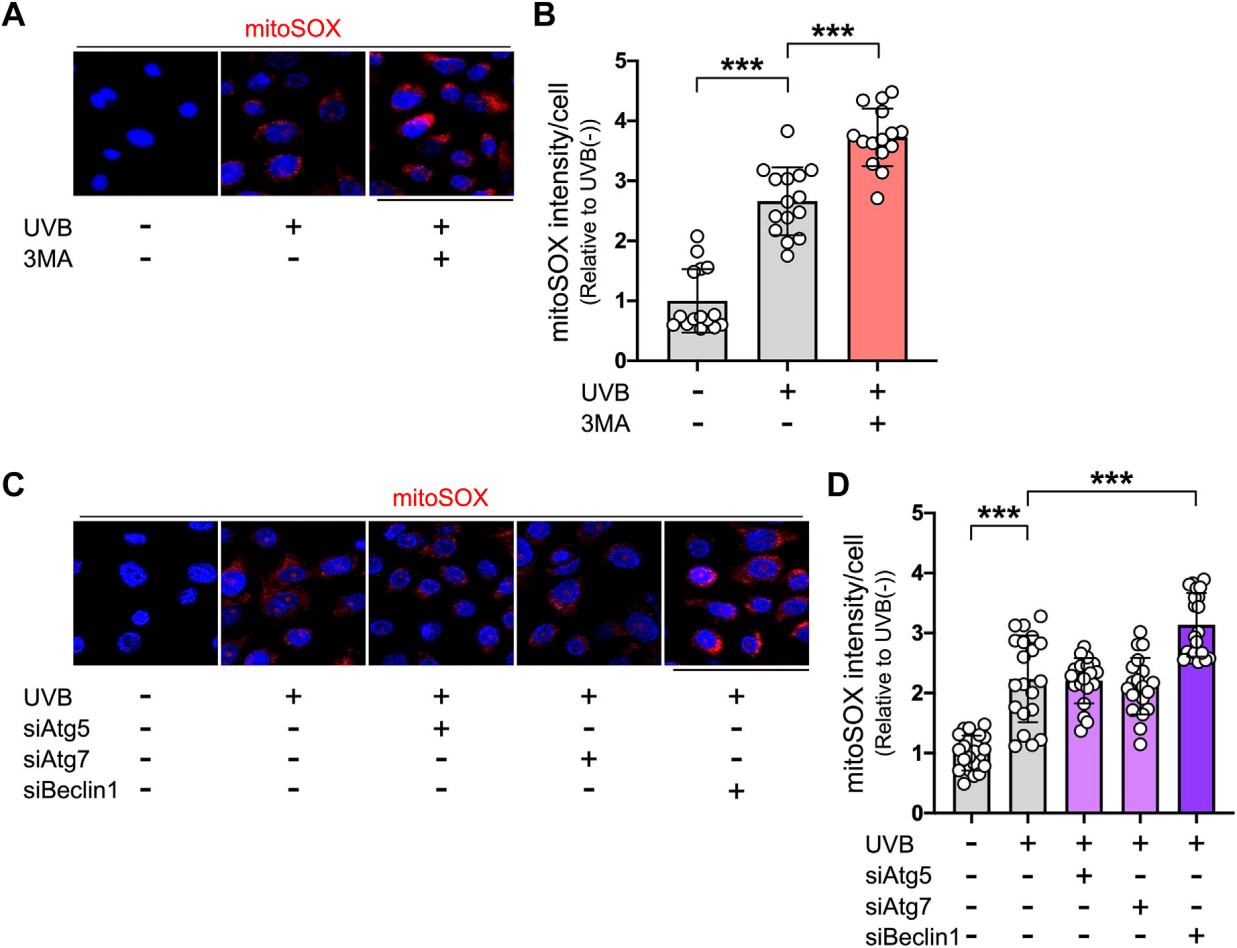
**Accumulation of damaged mitochondria in UVB-irradiated human keratinocytes is promoted under inhibition of alternative autophagy.***A*, representative staining with mitoSOX (*red*), an indicator of mitochondrial ROS, in keratinocytes, which were incubated for 24 h in the absence or presence of 3MA, then irradiated or not irradiated with UVB, and further cultured for 18 h. *B*, the intensity of mitoSOX staining in keratinocytes. Cells were counted and averaged across 15 randomly selected images in each experiment. *n* = 15 in each group. Repeated three independent experiments were performed. *C*, representative staining of mitoSOX (*red*) in keratinocytes, which were transfected with control, Atg5, Atg7, or Beclin1 siRNA for 48 h, then irradiated or not irradiated with UVB, and further cultured for 18 h. *D*, the intensity of mitoSOX staining in keratinocytes. Cells were counted and averaged across 20 randomly selected images in each experiment. *n* = 20 in each group. Repeated three independent experiments were performed. Nuclei were stained with Hoechst (*blue*). All data are expressed as the mean ± SD. ∗∗∗ *p* < 0.001. Scale bar represents 100 μm. UVB, ultraviolet B.

Increasing evidence suggests that mitochondrial damage and oxidative stress trigger NLRP3 inflammasome activation (33). Therefore, we sought to determine whether damaged mitochondria are generated by UVB radiation in keratinocytes and whether their accumulation is promoted when alternative autophagy is inhibited. As shown in Figure 3, *A* and *B*, the intensity of mitoSOX signals, an indicator of mitochondrial reactive oxygen species (ROS), was increased in UVB-irradiated keratinocytes, and this intensity was greatly increased in the presence of 3MA. Consistent with our data on UVB-induced IL-1β production, knockdown of Beclin1, but not knockdown of either Atg5 or Atg7, resulted in an increase in the intensity of mitoSOX signals in UVB-irradiated keratinocytes (Fig. 3, *C* and *D*), indicating that UVB-induced damaged mitochondria are accumulated in keratinocytes when alternative autophagy is inhibited.

**Figure 4:**
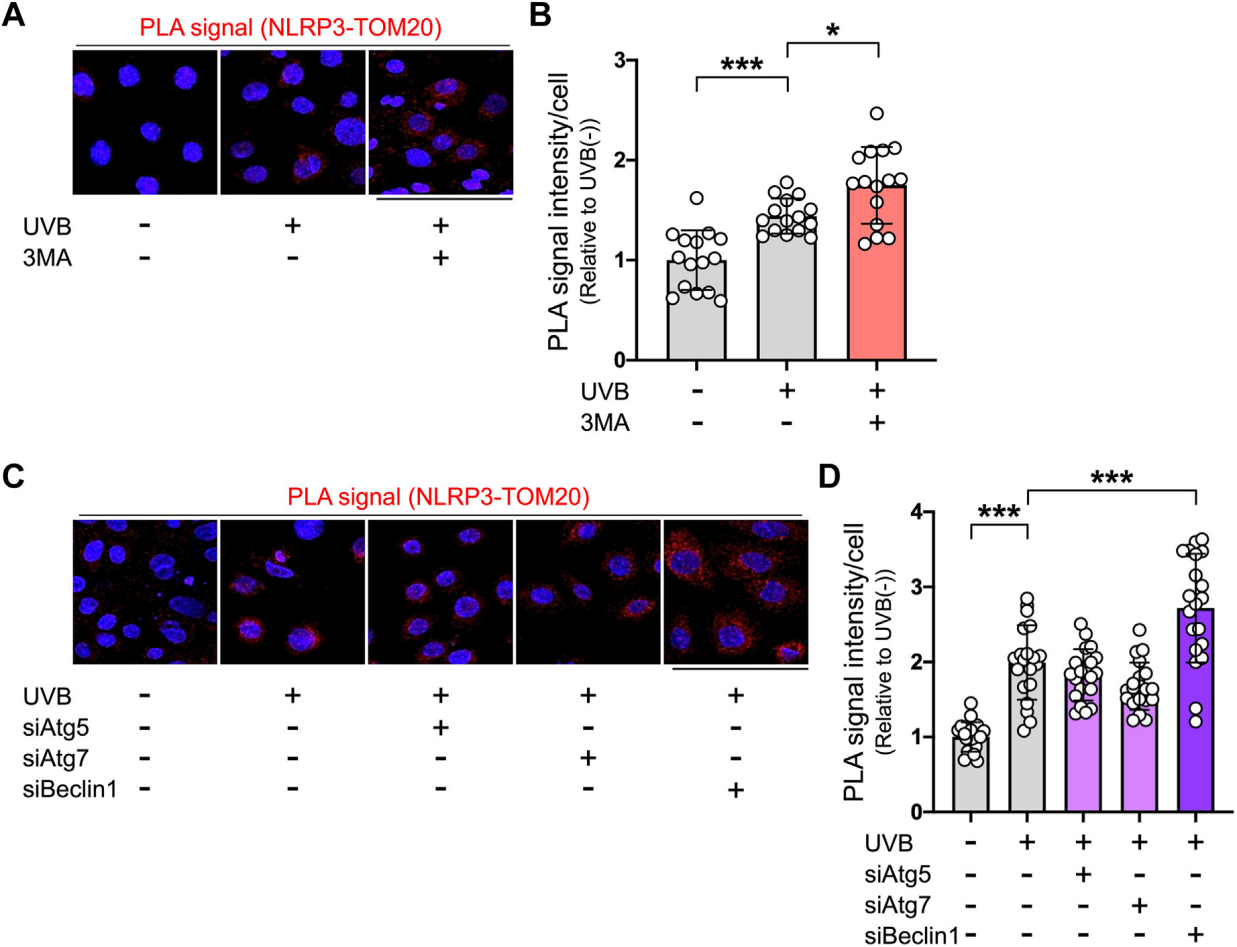
**NLRP3 co-localizes with UVB-induced damaged mitochondria in human keratinocytes.***A*, representative PLA staining (*red*), which detects protein complexes between NLRP3 and TOM20, in keratinocytes, which were incubated for 24 h in the absence or presence of 3MA, then irradiated or not irradiated with UVB, and further cultured for 18 h. *B*, the intensity of PLA signals in keratinocytes. Cells were counted and averaged across 15 randomly selected images in each experiment. *n* = 15 in each group. Repeated three independent experiments were performed. *C*, representative PLA staining (*red*) in keratinocytes, which were transfected with control, Atg5, Atg7, or Beclin1 siRNA for 48 h, then irradiated or not irradiated with UVB, and further cultured for 18 h. *D*, the intensity of PLA signals in keratinocytes. Cells were counted and averaged across 20 randomly selected images in each experiment. *n* = 20 in each group. Repeated three independent experiments were performed. Nuclei were stained with Hoechst (*blue*). All data are expressed as the mean ± SD. ∗ *p* < 0.05 and ∗∗∗ *p* < 0.001. Scale bar represents 100 μm. UVB, ultraviolet B.

Next, to determine whether UVB-damaged mitochondria are recognized by NLRP3, we visualized the protein–protein interaction between NLRP3 and mitochondrial outer membrane protein TOM20 in keratinocytes by means of *in situ* proximity ligation assay (PLA). Indeed, PLA signals were observed in UVB-irradiated keratinocytes, and these signals were markedly increased in the presence of 3MA (Fig. 4, *A* and *B*). Further, in accordance with the mitochondrial ROS data, knockdown of Beclin1, but not knockdown of either Atg5 or Atg7, resulted in a significant increase of PLA signals in UVB-irradiated keratinocytes (Fig. 4, *C* and *D*). These findings suggest that UVB-damaged mitochondria in human keratinocytes are recognized by NLRP3 but are eliminated in part by alternative autophagy, which thereby blocks NLRP3 inflammasome activation.

## Discussion

Our findings here indicate that Atg5/Atg7-independent alternative autophagy, rather than conventional autophagy, negatively regulates UVB-induced NLRP3 inflammasome activation in human keratinocytes by mediating the clearance of damaged mitochondria, thereby partly inhibiting NLRP3 inflammasome activation, and illustrates a previously unknown involvement of alternative autophagy in inflammation.

NLRP3 inflammasomes are a key activator of sterile inflammation, and their activation occurs in an all-or-none manner (34). Therefore, the cell must contain machinery to negatively regulate their activation in order to avoid excessive and long-lasting inflammasome activation. Although previous work was not carried out with skin, several studies have clarified that Atg5/Atg7-dependent conventional autophagy inhibits NLRP3 inflammasome activation through the degradation of its components and also by sensing targets, such as damaged mitochondria, and serves to prevent aberrant inflammation in some pathological conditions (35, 36, 37). Recent paper shows that Beclin 2 participates in the degradation of NLRP3 through LC3/Beclin1-independent autophagic pathway (38). Sunburn is a cutaneous sterile inflammation instigated by UVB-induced NLRP3 inflammasomes in keratinocytes (14, 39), and we have shown that UVB-induced nuclear DNA damage triggers NLRP3 inflammasome activation in keratinocytes (15). The results of our pharmacological approach in the present study show that the overall autophagy machinery negatively regulates UVB-induced NLRP3 inflammasome activation in keratinocytes; however, unexpectedly, we did not observe any significant effect of conventional Atg5/Atg7-dependent autophagy on UVB-induced NLRP3 inflammasome activation in keratinocytes. Conventional autophagy may have different roles in different cell types under different stresses and may even be inhibited by NLRP3 inflammasomes in some cases (40). Autophagy controls cellular homeostasis via at least two different pathways: conventional autophagy operates at basal levels and is induced under nutrient starvation or during cellular differentiation, whereas alternative autophagy can be induced by genotoxic stress (26). Our present results suggest that the suppression of UVB-induced NLRP3 inflammasome activation is caused in part by Atg5/Atg7-independent alternative autophagy in human keratinocytes.

The inflammasome sensor NLRP1 also highlights the importance of the link of various cellular stress with inflammatory responses in human keratinocytes (41, 42, 43, 44, 45, 46, 47). CRISPR/Cas9-mediated NLRP1 knockout approach reveals that NLRP1 inflammasome is essential for UVB-induced IL-1β production from human keratinocytes (43, 44). However, we do not observe the influence of siRNA-mediated knockdown of NLRP1 in 3MA-mediated IL-1β production from UVB-irradiated human keratinocytes under our experimental condition. The previous reports show the monitoring of NLRP1 inflammasome-mediated IL-1β production from keratinocytes irradiated with high dose of UVB (86.4 or 100 mJ/cm^2^) (43, 44). On the other hand, we examine changes of NLRP3 inflammasome-mediated IL-1β production from keratinocytes irradiated with low dose of UVB (20 mJ/cm^2^), which is equivalent to sunlight exposure during normal daily activities (48). It has been reported that keratinocytes underwent necrotic cell death with high dose of UVB (80 mJ/cm^2^), and cellular responses to different doses of UVB radiation are altered (49). Therefore, we speculate that NLRP1 and NLRP3 are required for UVB-induced inflammasome activation in human keratinocytes under different cellular responses in UVB dose-dependent manner. However, it is highly desirable to monitor proper inflammasome-mediated IL-1β production while avoiding necrotic cell death–mediated passive IL-1β release following high dose UVB radiation, but this requires future explorations. In addition, our data show that the blockade of NLRP3 significantly suppresses UVB-induced IL-1β production from human keratinocytes, but not entirely. Therefore, we speculate that previously uncharacterized factor(s), such as other NLR members or caspase-4– and caspase-5–mediated non-canonical inflammasome, may also regulate UVB-induced inflammasome activation in human keratinocytes.

It remains technically challenging to monitor and characterize alternative autophagy, but Ulk1^746^ phosphorylation was recently identified as a suitable marker (31). Here, we found that Ulk1^746^ phosphorylation is induced by UVB radiation in keratinocytes. Genotoxic stress, such as exposure to DNA-damaging chemotherapeutic agents, induces alternative autophagy (31), and UVB in sunlight also causes genotoxic stress by directly damaging DNA (2). Our data suggests that UVB-induced alternative autophagy in keratinocytes is triggered at least in part by DNA damage. As DNA damage is associated with photoaging, mutagenesis, and skin carcinogenesis, cells have evolved several DNA repair machineries (50). Unrepaired DNA damage can result in pathological levels of nucleus-to-mitochondria signaling, which can cause mitochondrial dysfunction (51). We observed that damaged mitochondria are accumulated in UVB-irradiated keratinocytes, and concurrently NLRP3 co-localizes with the mitochondrial outer membrane under inhibition of alterative autophagy. These results suggest that UVB-induced damaged mitochondria activate NLRP3 inflammasomes in keratinocytes, while alternative autophagy mediates the clearance of damaged mitochondria to restore homeostasis.

Mitochondrial dysfunction is accompanied by impairment of ATP synthesis, increased ROS generation, and calcium imbalance and consequently is a hallmark of many diseases, including skin disorders such as aging and cancer (52). Healthy and functional mitochondria are subject to multiple quality control processes, including clearance of damaged mitochondria through a selective form of autophagy, coined mitophagy, to modulate their function in response to physiological adaptations and stress conditions (53). Intriguingly, alternative autophagy predominantly participates in the removal of unwanted mitochondria in mammalian cells (54, 55) and notably occurs during dynamic metabolic reprogramming and genotoxic stress, such as development of erythrocytes (23), reprogramming of induced pluripotent stem cells (56), and chemotherapeutic drug treatment (31). Consistent with these insights, our findings indicate that alternative autophagy may also play a key role in protecting the skin against genotoxic stress due to sunlight-induced DNA damage. In marked contrast, Atg7-dependent conventional autophagy in the epidermis exacerbates UVB-induced inflammatory responses and skin tumorigenesis (57). However, the mechanism of alternative autophagy-mediated mitochondria clearance has not been fully elucidated. In order to further elucidate the precise pathway of Atg5/Atg7-independent alternative mitophagy, the identification of specific cargo receptor for recognition of damaged mitochondria is required, which will provide insights into the mechanism of alternative mitophagy.

Melanocytes in the skin are generally long-lived cells (58) and are therefore continuously exposed to large amounts of UV radiation from sunlight, leading to the accumulation of pathogenic mutations known to drive melanoma (59). Melanocytes can also mediate the UV-induced inflammatory responses along with keratinocytes (60). Therefore, it is also important for future studies to investigate the protective role of alternative autophagy in melanocytes as well as keratinocytes in UV-exposed skin.

In conclusion, our findings indicate a protective role of alternative autophagy against UVB-induced NLRP3 inflammasome activation in human keratinocytes through the clearance of damaged mitochondria and have an implication for understanding the intracellular machinery through which epidermal homeostasis is restored after UV radiation. Alternative autophagy may thus serve to restore skin homeostasis and might be a new therapeutic target for treating sunburn and associated cutaneous disorders.

## Experimental procedures

### Cell culture

Neonatal foreskin normal human epidermal keratinocytes were obtained from Kurabo. Serum-free keratinocyte growth medium containing low calcium (0.06 mM), bovine pituitary extract and epidermal growth factor, insulin, hydrocortisone, and antibiotics (gentamicin/amphotericin) were purchased from Gibco. Cells were seeded at a density of 3 to 5 × 10^4^ cells/cm^2^ into 75 cm^2^ cell culture flasks and cultured at 37 °C under an atmosphere of 5% CO_2_ in air. Third or fourth passage cells were used for the experiments. Cells were treated with 1 mM 3MA (3977; R&D Systems), 100 nM MCC950 (86428; Cell Signaling Technology), 500 nM rapamycin (BML-A275-0005; Enzo Life Sciences), 500 nM SAR405 (5.33063; Merck), or 10 μM SBI-0206965 (18477; Cayman Chemical) for 24 h before UVB radiation and then cultured in the presence of 3MA, MCC950, rapamycin, SAR405, or SBI-0206965.

### UVB radiation

Subconfluent cells were irradiated through PBS with 20 mJ/cm^2^ UVB generated by a broad-spectrum lamp (TL20W/12RS; Philips). Radiation intensity was measured using a UV radiometer (UVR-3036/S2; Topcon).

### RNA interference

siRNAs against Atg5 (Stealth HSS114103), Atg7 (Stealth HSS116182), Beclin1 (Stealth HSS189498), NLRP1 (Stealth HSS117757), NLRP3 (Stealth HSS132811 and Stealth HSS132812), Rab9 (Stealth HSS113896), and Wipi3 (Stealth HSS183480) were purchased from Invitrogen. A non-targeting scrambled siRNA (Invitrogen) with a similar GC nucleobase content was used as the control in this experiment. Each siRNA (200 nM) was transfected into cells (6–9 × 10^5^ cells) by using an Amaxa Human Keratinocyte Nucleofector Kit (Lonza), according to the manufacturer’s protocol.

### Western blot analysis

Cells were lysed on ice in PhosphoSafe extraction buffer (Merck), including protease inhibitor (Nacalai Tesque). After centrifugation, supernatants were mixed with NuPAGE LDS sample buffer (Invitrogen) and loaded onto Bis-Tris gels. After electrophoresis, the proteins were blotted onto PVDF membranes. The membranes were blocked and incubated with primary antibodies against Atg5 (1:1000, 12994; Cell Signaling Technology), Atg7 (1:1000, 8558; Cell Signaling Technology), β-actin (1:1000, sc-47778; Santa Cruz), Beclin1 (1:1000, 3495; Cell Signaling Technology), light chain 3B (1:1000, 3868; Cell Signaling Technology), NLRP1 (1:1000, AG-25B-0005; AdipoGen Life Sciences), NLRP3 (1:1000, 13158; Cell Signaling Technology), Rab9 (1:1000, 5133; Cell Signaling Technology), Ulk1 (1:1000, 8054; Cell Signaling Technology), and Wipi3 (1:1000, PA5-50864; Thermo Fisher Scientific), and corresponding secondary antibodies labeled with alkaline phosphatase, and then visualized with a Novex AP Chemiluminescent Detection Kit (Invitrogen), using a Fusion FX (Vilber Lourmat). Band intensities were analyzed using Image J software (https://imagej.net/ij/). The experiments were conducted in triplicate for siRNA-mediated knockdown tests or quadruplicate for p-Ulk1^746^ expression test.

### Immunoprecipitation

Cell lysates were incubated with p-Ulk1^746^ antibody (31) for 60 min at 4 °C. The antibody-bound target protein complexes were eluted with a Capturem IP & Co-IP Kit (Takara Bio), according to the manufacturer’s protocol.

### Measurement of autophagic flux

Cells were stained with Cyto-ID autophagy dye (Enzo Life Sciences) for 30 min at 37 °C. Cyto-ID fluorescence was analyzed using a flow cytometer (FACS Canto).

### Measurement of cytokines

After treatment, the culture medium was collected and centrifuged. IL-1β in the supernatant was analyzed using a Human IL-1β Quantikine ELISA Kit (R&D Systems). IL-6 in the supernatant was analyzed using a Human IL-6 Quantikine ELISA Kit (R&D Systems).

### Immunocytochemical staining

Cells were fixed with 4% paraformaldehyde in 5 min at room temperature, permeabilized with 20 μg/ml digitonin in PBS in 2 min at room temperature, and then blocked with 5% normal goat serum in PBS for 30 min at room temperature. Cells were treated with primary antibody against cyclobutane pyrimidine dimer (1:500, NM-DND-001; Cosmo Bio), GS28 (1:200, 611184; BD Biosciences), p-H2AX^139^ (1:400, 9718; Cell Signaling Technology), and p-Ulk1^746^ (1:200) at 4 °C overnight and then with corresponding secondary antibodies labeled with Alexa Fluor 488 and 594. Nuclear counterstaining was done with Hoechst 33342 Ready Flow Reagent (Thermo Fisher Scientific). The stained cells were imaged with a ZEISS confocal microscope.

### Measurement of mitochondrial ROS

MitoSOX (Invitrogen) was used to detect mitochondrial ROS generation according to the manufacturer’s instructions. Cells were incubated with mitoSOX (10 μM) at 37 °C for 20 min. Labeled cells were washed with PBS three times and visualized under the ZEISS confocal microscope.

### Proximity ligation assay

PLA was performed in 4% paraformaldehyde-fixed cells. Cells were incubated with NLRP3 (1:200, AG-20B-0014; AdipoGen Life Sciences) and TOM20 (1:200, sc-11415; Santa Cruz Biotechnology) primary antibodies to detect specific interaction between NLRP3 and TOM20. Secondary antibodies conjugated with oligonucleotides, ligation solution, and amplification solution together with polymerase were added successively by using a Duolink *In Situ* Kit (Sigma-Aldrich) according to the manufacturer’s protocol.

### Statistical analysis

All data are expressed as the mean ± SD. Statistical significance in two-group comparisons was determined with two-tailed Mann-Whitney *U* test. Statistical significance in multiple-group comparisons was determined with one-way ANOVA with Bonferroni’s multiple comparisons test. Statistical analyses were performed by Prism 8 (GraphPad). *p* value < 0.05 was considered significant.

## Data availability

No datasets were generated or analyzed during the current study.

## Supporting information

This article contains supporting information.

## Conflicts of interest

The authors declare that they have no conflicts of interest with the contents of this article.

## Supporting information

Supporting Figures S1–S5 Captions
