# Supplementary material for: Alternative autophagy dampens UVB-induced NLRP3 inflammasome activation in human keratinocytes
Source: J Biol Chem. 2024 Mar 17;300(4):107173. doi: 10.1016/j.jbc.2024.107173 (PMC11002869; doi:10.1016/j.jbc.2024.107173)
Supplement: Supporting Figures S1–S5 Captions [file mmc1.docx]

Supplemental Figures

**Alternative autophagy dampens UVB-induced NLRP3 inflammasome activation in human keratinocytes**

Tatsuya Hasegawa^1,^*, Saori Noguchi^2^, Masaya Nakashima^1^, Masashi Miyai^1^, Makiko Goto^1^, Yuko Matsumoto^1^, Satoru Torii^2^, Shinya Honda^2^, and Shigeomi Shimizu^2^

* Corresponding authors:

Email: [tatsuya.hasegawa@shiseido.com](mailto:tatsuya.hasegawa@shiseido.com)

**This file includes:**

Supplementary Figures S1 to S5

**SUPPLEMENTARY FIGURES**

**Supplementary Figure S1. Autophagy inhibitors promote UVB-induced IL-1β** **production in human keratinocytes.** (**A**) Quantification of IL-1β production by ELISA in the supernatants of keratinocytes, which were incubated for 24 h in the absence or presence of autophagy inhibitor SAR405, and then irradiated or not irradiated with UVB, and further cultured for 48 h. (**B**) Quantification of IL-1β production by ELISA in the supernatants of keratinocytes, which were incubated for 24 h in the absence or presence of autophagy inhibitor SBI (SBI-0206965), and then irradiated or not irradiated with UVB, and further cultured for 48 h. All data are expressed as the mean ± standard deviation. *n* = 4 per group. *** *P* < 0.001.

**Supplementary Figure S2. Autophagy inhibitors promote UVB-induced NLRP3 inflammasome activation in human keratinocytes.** (**A**) Quantification of IL-1β production by ELISA in the supernatants of keratinocytes incubated for 24 h in the absence or presence of SAR405 or MCC950, and then irradiated or not irradiated with UVB, and further cultured for 48 h (*n* = 6 per group). (**B**) Quantification of IL-1β production by ELISA in the supernatants of keratinocytes incubated for 24 h in the absence or presence of SBI (SBI-0206965) or MCC950, and then irradiated or not irradiated with UVB, and further cultured for 48 h (*n* = 5 per group). All data are expressed as the mean ± standard deviation. *** *P* < 0.001.

**Supplementary Figure S3. Atg5/Atg7-dependent conventional autophagy does not suppress UVB-induced IL-6 production in human keratinocytes.** (**A**) Quantification of IL-6 production by ELISA in the supernatants of keratinocytes transfected with control siRNA or Atg5 siRNA, then irradiated or not with UVB, and further cultured for 48 h. (**B**) Quantification of IL-6 production by ELISA in the supernatants of keratinocytes transfected with control siRNA or Atg7 siRNA, then irradiated or not with UVB, and further cultured for 48 h. All data are expressed as the mean ± standard deviation. *n* = 3 per group. *** *P* < 0.001, ns: not significant.

**Supplementary Figure S4. UVB radiation induces DNA damage in human keratinocytes.** (**A**) Representative immunocytochemistry of cyclobutane pyrimidine dimer (CPD) (green) and γH2AX (red) in keratinocytes, which had been irradiated or not with UVB, and further cultured for 6 h. Blue: Hoechst nuclear stain. (**B**, **C**) The percentage of CPD^+^ cells (**B**) or γH2AX^+^ cells (**C**) in keratinocytes. *n* = 15 in each group. All data are expressed as the mean ± standard deviation. *** *P* < 0.001. Scale bar: 100 μm.

**Supplementary Figure S5. Knockdown of Wipi3 promotes UVB-induced IL-1β** **production in human keratinocytes.** (**A**) Representative example showing the expression level of Wipi3 determined by western blotting in cell lysates of keratinocytes transfected with either control siRNA or Wipi3 siRNA. (**B**) Quantification by ELISA of IL-1β in the supernatants of keratinocytes that were transfected with control siRNA or Wipi3 siRNA for 48 h, and then irradiated or not irradiated with UVB, and further cultured for 48 h (*n* = 5 per group). All data are expressed as the mean ± standard deviation. * *P* < 0.05 and *** *P* < 0.001.
